# Southern Dental Association

**Published:** 1890-08

**Authors:** 


### EDITORIAL.

### Southern Dental Association.

The annual meeting of the Southern Dental Association was held in Atlanta, Georgia, on the 15th of July and three subsequent days.

There was quite a large attendance of both the membership and visitors. The meeting was an enthusiastic and interesting one. The time was very closely occupied in the ligitimate work of the body. A large number of excellent papers were read and most of them discussed. During the latter part of the meeting the discussion of papers was omitted, there being so many papers as to render discussion impracticable. And during the closing hours of the meeting a number of papers were read by title. There was throughout the utmost harmony in the work of the association.

There seems to be little or no desire for preferment amongst the members, the aim being in the selection of officers to secure those who will be efficient and energetic in preparation for the meetings and carrying them on while in progress. We have never seen the time better utilized than in these meetings; indeed, there was no time wasted. This was due, in a large measure, to the promptness, energy and efficiency of the presiding officer, Dr. John C. Storey, of Dallas, Texas. The doctor knows well how to guide a body like this in order to secure the largest amount of work.

The association took the initiative steps with reference to two very important matters. The one was the appointment of a committee of five persons to confer with the committee of like number appointed by the American Dental Association in reference to holding a grand meeting of the profession of this country to which the dentists of all other countries should be invited, during the time of the Great Columbian Exposition to be held in Chicago in 1893. And if the same harmony and concurrence is manifested by the profession generally as was there shown a grand success will be achieved. The other matter taken up, considered and a committee appointed to take the matter in charge, was that of securing a permanent home for the Association at some desirable point in the mountains of Tennessee or North Carolina where should be established a kind of Dental Institute that should continue in operation from two to four weeks (in other words, a Dental Chatauqua) where dentists could go for their vacation and enjoy social amenities combined with as much professional work as they might choose.

Doctor M. W. Richards, of Knoxville, Tenn., champion of this movement, which, when fully explained by the doctor, received the unqualified approbation of all the members present. So far as our experience goes, this was the best meeting of this association ever held, and if the interest there shown is maintained in the future, the Southern Dental Association will be a great agency for promoting the interests of the profession not only in the South but throughout the country.

The next number of the REGISTER will contain a brief report of the proceedings of the association.

---

At the meeting of the Michigan Dental Society the principal points in Dr. Crouse's remarks were: First, that by combining in some such an organization as the Dental Protective Association we band the strength of ten thousand men into one. All defence necessary can be made with but little more expense than would be required for an individual. The preparation of one case will answer for all and all evidence can be collected better by an association than in any other way.

Second, that it is very important that all get into the association at this time for the reason that an appealed suit to the Supreme Court before the association was formed, will probably be decided in favor of the Crown Co., owing to deficiency of evidence and imperfect presentation. If the Crown Co. win this suit they will send notices all over the U. S., thereby demoralizing the profession and causing many to pay them money who should be in the Protective Association and avoid that calamity. The Protective Association will be ready with new evidence and a new record and can take care of its members against any claims of the Crown Co. If you are not in the association the association will not take care of you when the time comes.

If each man in the profession will pay $10 and assume a responsibility of ten more without any further assessments, we will have an organization that is sure to break up all this abuse and save the dental profession an annual outlay of, on an average, of $100 each to unjust claimants. Remember it will cost more than $10 later to join, and will certainly cost more than that if you do not protect yourself.

